# How the obsession to eat healthy food meets with the willingness to do sports: the motivational background of orthorexia nervosa

**DOI:** 10.1007/s40519-019-00642-7

**Published:** 2019-02-06

**Authors:** Márton Kiss-Leizer, István Tóth-Király, Adrien Rigó

**Affiliations:** 1 Institute of Clinical Psychology, Semmelweis University, Budapest, Hungary; 2 School of Psychology, ELTE Eötvös Loránd University, Budapest, Hungary; 3 Institute of Psychology, ELTE Eötvös Loránd University, Izabella utca 46, Budapest, 1064 Hungary

**Keywords:** Orthorexia nervosa, Eating disorders, Sport, Health anxiety

## Abstract

**Purpose:**

Given its relevance, the present study sought to reveal the motivational background of orthorexia nervosa (ON) and to examine its association to do sports.

**Methods:**

A total number of 739 participants completed a self-administered, online questionnaire including questions related to sports and three scales: Ortho-11-Hu, Motivation for Healthy Behaviors in Orthorexia Nervosa Questionnaire (MHBONQ) and Short Health Anxiety Inventory (SHAI). The age of the respondents ranged from 18 to 72 years (*M* = 29.67, SD = 10.18) and 79.16% of them were female. The majority of the subjects trained 3–4 times a week (37.2%), usually for 1–2 h per week (25.8%).

**Results:**

According to the hierarchical multiple regression analysis, social desirability, guilt over skipping training and health anxiety were the strongest predictors of ON with explaining 46% of the variance of ON.

**Discussion:**

The results of the present study suggested that obsessive features of sport activities (guilt over skipping training, counting calories during training) play an important role in ON. People with a higher level of ON tend to reach other people’s respect, protect their general health and regulate negative emotional states through healthy eating.

**Level of evidence:**

Level V, descriptive cross-sectional study.

**Electronic supplementary material:**

The online version of this article (10.1007/s40519-019-00642-7) contains supplementary material, which is available to authorized users.

## Introduction

The different manuals of mental disorders may vary from time to time: certain disorders are getting removed from the category of mental illnesses, while others are getting an increasing social and scientific interest by being added to the nosological system. The reconstruction is often related to the actual socio-cultural influences, which is particularly true in the case of eating disorders [1]. There is a growing focus on healthy eating in the Western culture, and in the past 2 decades—besides the ideal of thinness—the ideal of being healthy has become more and more important [2].

The obsession with healthy food and proper nutrition has been described by Bratman [2] who defined orthorexia nervosa (ON) as an eating disorder, characterized by an excessive preoccupation with eating healthy food. Individuals with ON have self-defined dietary restrictions and only consume food that they consider healthy. Although Dunn and Bratman [3] proposed diagnostic criteria of ON, there is no uniform description or any standardized criteria for ON. ON is not in the medically approved nosology systems (The Diagnostic and Statistical Manual of Mental Disorders, Fifth Edition-DSM-5 [4], International Statistical Classification of Diseases and Related Health Problems 11th Revision—ICD-11) [5]; however, there are some suggestions that the DSM-5 should modify the category of EDNOS (Eating disorders not otherwise specified), and define new types of eating disorders [6].

The definition of ON mostly focuses on eating habits, and does not refer to other characteristics of healthy lifestyle that can be indicative of ON or can help differentiate between ON and healthier ways of following pure diet. For example, regular sport activities play an unambiguously important role in creating a healthy life, including proper weight management, efficient heart functioning, lower cholesterol level, hypertension, stress and depression levels [7–9], but its excessive forms are connected to different eating disorders [e.g., anorexia nervosa (AN), bulimia nervosa (BN), and EDNOS] [10]. In a study, 45.5% of the participants with eating disorders were compulsive exercisers with the highest prevalence being for the restrictive subtype of AN [9]. The persistence of such behavior is correlated with more severe psychopathology, worse treatment outcome, and higher risk of relapse. Patients with eating disorders are more likely to do intensive physical exercise to regulate negative emotional states instead of being trained or to improve general health [10].

The potential role of exercise is not included in the criteria of ON [2, 3] and only a few studies investigated the possible impact of sport activities on ON [6, 11, 12]. More frequent exercising was associated with higher ON values in women, while the stronger internalization of ideal muscular body resulted in higher ON tendency in men [13]. Higher ON tendency is not only associated with the choice of healthy food (consuming more vegetables, fruits and full grains), but also with healthier lifestyle habits such as doing more sports or drinking less alcohol [6, 12]. In case of striving for healthy nutrition, more frequent (but moderated) physical activity may reflect the motivation to reach a healthy lifestyle. However, in line with other eating disorders, it is possible that exercise, due to its special characteristics, might be considered as a symptom of ON, with the same underlying pathological background as the unhealthy way of healthy eating. In case of ON, there is no such differentiated data about the possible role of exercise.

Certain groups, professions, and people doing some specific activities (ashtanga yoga practitioners, performance artists, or athletes) have been shown to have increased risk of ON [13–16]. Many athletes choose strictly regulated dietary habits, which can easily lead to eating disorders [13, 14, 16]. In a recent study on problematic eating, 157 athletes were matched with a control group of 217 respondents, the former group was characterized by significantly higher ON tendency [16]. Moreover, ON together with high level of physical activity has been found more frequent in men studying sport than women in business studies [17], and a positive correlation has been identified between ON and exercise addiction in the members of fitness studios [18]. ON symptoms were found to be stronger in individuals who perceived themselves muscular and thin [19]. Subjects with ON symptomatology initially do sports to improve their general health, but later this can lead to exercise addiction, rigid schedules of sport activities, injury and illness [20]. Overall, the relationship between ON and exercise appears to be very complex.

In a more comprehensive understanding of physical activity and healthy eating (and their excessive forms), the key might be to explore the motivational background of these behaviors. Both physical exercise and healthy nutrition can be framed within a desire to reach a healthier way of life, which is culturally accepted, legalized and even expected, but both might have such motivational backgrounds that can turn to a more pathological direction. Regarding the motivational background of excessive physical activity, many potential explanatory factors have been identified: to regulate negative emotional affects [10], to change shape and lose weight [9], to compensate binge eating [21] or the desire to be muscular [22]. Similarly, some background factors have been explored behind (pathological) healthy eating: to improve general health, to overcome a chronic disease [2], to feel satisfied about the self-defined eating habits, to reach a better social position [3], to get spiritual completeness [23], to regulate emotions [24], or to compensate unhealthy eating [25]. Overall, it appears that one’s motivation for healthy behavior might be an important predictor of ON, yet it remains a relatively understudied area.

Based on previous studies [13, 14, 16–18], we expected that among people with high ON tendency, sport activities play a prominent role, which manifests in more regular and longer exercise sessions. We also expected that people with high ON tendencies do exercise in a more obsessive way than others. The other purpose of the present study was to explore the motivational background of ON, and to investigate the similarities of the background factors of excessive exercise and ON.

## Methods

### Participants and procedure

The present study was approved by the Research Ethics Committee of Eötvös Loránd University, Budapest, Hungary and was conducted in accordance with the Declaration of Helsinki. Participants were recruited from social media websites specialized in dietary habits (e.g., healthy lifestyle, healthy nutrition, exercise promoting sites). The questionnaire was also advertised by fitness trainers and lifestyle consultants. Participants were informed about the general aim and the topic of the study. If they wished to participate, they had to check a box; otherwise, they were excluded. The study was carried out with the explicit consent of the participants.

Altogether, 739 respondents (79.16% female) were recruited in the present study. Their age ranged from 18 to 72 years (*M* = 29.67 years, SD = 10.18). With respect to the level of education, university graduates were represented in the highest proportion (52.2%), the second largest group was high school graduates (44.5%). The “other” category was marked by 2.8% of the sample and only 3 responders had primary education (0.4%). Based on self-reported membership of risk groups, 11.5% of the participants were athletes, 4.3% of them were performance artists, and 4.7% of them were ashtanga yoga practitioners. A high proportion of respondents in the sample did sports; only 104 participants did not do exercise at all. The majority of the respondents had trainings 3–4 times a week (37.2%); 25.8% of the participants trained for 1–2 h weekly, nearly 25% of them for 3–4 h and 35.2% for 5 or more than 5 h. The average body mass index (BMI) value of the participants was 22.85 (SD = 4.02).

### Measures

*Ortho-11-Hu* To measure the tendency to ON, we used Ortho-11-Hu [3], which is the Hungarian version of ORTO-15 by Donini et al. [26]. The scale is based on Bratman’s [2] original questionnaire, but it is supplemented with few items about obsessive–compulsive traits from the Minnesota Multiphasic Personality Inventory [27]. The original ORTO-15 is a self-reported questionnaire containing 15 questions about the person’s attitude towards healthy eating. The Hungarian version has been shortened to 11 items and previously demonstrated adequate factor structure and internal consistency [6]. Respondents indicated their answers on a four-point Likert scale (1 = always, 4 = never); lower values indicate a higher tendency to ON.

*Sport habits* Participants were asked to report their sports habits: when and how often they are doing sport and how many hours they spend on sport activities on an average week. We added two further questions about sports to measure the possible obsessive features: “Do you feel guilty if you skip a training?” “Do you monitor how many calories have you burnt during the exercise?”. Participants were able to respond on a four-point scale (1 = always; 4 = never).

*Motivation for Healthy Behaviors in Orthorexia Nervosa Questionnaire* (*MHBONQ*). To assess participants’ underlying motivations for healthy behaviors, an eight-item questionnaire was constructed for the purpose of the present study including four factors: mood modification (i.e., monitor health in a negative affective state), weight control (i.e., being healthy to achieve desired weight), social desirability (i.e., being healthy to gain social support), and healthy lifestyle (i.e., being healthy has positive consequences). Participants indicated their answers on a four-point scale (1 = completely true; 4 = not true at all). More information is provided about the construction of the instrument and the final version in the online supplements for interested readers.

*Short Health Anxiety Inventory* (*SHAI*) To control the potential effect of health anxiety on ON, the 18-item SHAI was administered [28] assessing both normal health concerns and serious health anxiety. It is an appropriate scale for estimating the level of health anxiety, independently from the actual physical condition. Items pertain to health concerns, monitoring the changes of physical state, and the consequences of a possible disease. The Hungarian version demonstrated adequate factor structure and internal consistency [29].

### Statistical analysis

Information pertaining to the factor structure of the MHBONQ is available in the online supplements. Analyses were conducted in SPSS version 22 (IBM SPSS Inc., Chicago, Illinois). We first examined the descriptive statistics, the normality and the reliability of the variables of interest. We divided the sample into three different approximately equal groups on the basis of their Orto-11-Hu scores (Low risk: Ortho-11-Hu range of score from 35 to 43, *n* = 245, medium risk: Ortho-11-Hu range of score from 30 to 34, *n* = 260, high risk: Ortho-11-Hu range of score from 14 to 19, *n* = 234). After grouping the participants, one-way analysis of variance (ANOVA) with Bonferroni’s post-hoc test was used for group-based comparisons, Spearman’s rank-order correlation was used to assess the association between the variables, and hierarchical multiple regression was performed to investigate the associations of the predictor variables and orthorexia nervosa.

## Results

### Preliminary results

Descriptive statistics, normality and reliability indices are reported in Table 1. All variables had a normal distribution and acceptable internal consistencies. The three groups (high risk of ON 31.7%; moderate risk of ON 35.2%; low risk of ON 33.2% of the sample) were subsequently compared on the four items measuring sporting habits. Regarding the reported weekly occasions of training, there was a significant difference between the three groups, *F*(2,632) = 7.08, *p* < 0.01. The Bonferroni post-hoc test revealed that the high risk (*M* = 3.01, SD = 0.72, *p* < 0.01) and moderate risk (*M* = 3.10, SD = 0.81, *p* < 0.05) groups differed from the low-risk one (*M* = 3.30, SD = 0.80), but not from one another (*p* = 0.66 between moderate and high-risk groups). In the case of the hours spent with training, there was also a significant difference between the groups, *F*(2,632) = 3.50, *p* < 0.05; the high-risk group (*M* = 4.53, SD = 1.12) differed from the low-risk group (*M* = 4.71, SD = 1.17, *p* < 0.05), other differences were non-significant (*p* = 0.32 between high-risk and moderate risk as well as *p* = 0.83 between the moderate and low-risk groups). Differences also pertained to the item measuring being guilty when skipping training, *F*(2,736) = 78.73, *p* < 0.01, where all three groups differed from one another (*p* < 0.01 for all post-hoc tests): high risk (*M* = 2.20, SD = 0.88), moderate risk (*M* = 2.58, SD = 0.83), low risk (*M* = 3.16, SD = 0.93) (Higher value means less guilt). Finally, groups also had statistically significant differences regarding the attention they pay to the amount of burnt calories during training, *F*(2,736) = 56.43, *p* < 0.01. Similar to the previous results, all three groups differed from one another (once again, *p* < 0.01 for all post-hoc tests): high risk (*M* = 2.64, SD = 1.02), moderate risk (*M* = 3.12, SD = 0.96), low risk (*M* = 3.53, SD = 0.73). (Higher value means less attention to the amount of burnt calories.) Taken together, these results suggest that respondents belonging to the high-risk ON group, compared to the moderate and low-risk groups, spent the most amount of time with training, they felt guiltier if they skipped a training, and they paid more attention to the amount of calories they burnt during training.

**Table 1:** Descriptive statistics, normality and reliability indices

Variable	Range	Mean	Standard deviation	Skewness (SE)	Kurtosis (SE)	*α*
Orthorexia nervosa (Ortho-11-Hu)	1–4	2.88	0.50	− 0.56 (0.09)	0.14 (0.18)	0.82
Health anxiety (SHAI)	1–4	3.17	0.34	− 1.09 (0.09)	2.12 (0.18)	0.81
Mood modification (MHBONQ)	1–4	3.18	0.73	− 0.50 (0.09)	− 0.49 (0.18)	0.74
Weight control (MHBONQ)	1–4	1.92	0.71	0.70 (0.09)	0.63 (0.18)	0.65
Social desirability (MHBONQ)	1–4	2.94	0.85	− 0.38 (0.09)	− 0.68 (0.18)	0.80
Healthy lifestyle	1–4	1.37	0.57	1.78 (0.09)	3.71 (0.18)	0.81
Weekly occasions of training^a^	1–4	3.13	0.79	− 0.51 (0.10)	− 0.46 (0.19)	–
Hours spent with training^b^	1–6	4.69	1.15	− 0.57 (0.10)	− 0.34 (0.19)	–
Guilt if skipping training	1–-4	2.65	0.93	− 0.02 (0.09)	− 0.92 (0.18)	–
Calorie counting during training	1–4	3.10	0.98	− 0.72 (0.09)	− 0.65 (0.18)	–

*SE* standard error, *α* Cronbach’s alpha, *SHAI* Short Health Anxiety Inventory; *MHBONQ* Motivation for Healthy Behaviors in Orthorexia Nervosa Questionnaire

^a^1 = 7 occasions or more, 2 = 5–6 occasions, 3 = 3–4 occasions, 4 = 1–2 occasions

^b^1 = more than 20 h, 2 = 11–20 h, 3–7–10 h, 4 = 5–6 h, 5 = 3–4 h, 6–1–2 h

### Main results

Correlations between the variables are reported in Table 2. Orthorexia nervosa was moderately associated with health anxiety, the healthy behavior motivations, and the items measuring the “obsessive” aspects of training. On the other hand, weekly occasions of training and the hours spent with training were only weakly related to ON.

**Table 2:** Correlations between the examined variables

Variable	1	2	3	4	5	6	7	8	9
1. Orthorexia nervosa	–								
2. Health anxiety	0.31**	–							
3. Mood modification	0.34**	0.06	–						
4. Weight control	0.34**	0.08*	0.19**	–					
5. Social desirability	0.48**	0.12**	0.26**	0.32**	–				
6. Healthy lifestyle	0.38**	0.12**	0.21**	0.40**	0.27**	–			
7. Weekly occasions of training	0.16**	− 0.12**	0.23**	0.23**	0.13**	0.20**	–		
8. Hours spent with training	0.13**	− 0.03	0.27**	0.20**	0.08*	0.14**	0.68**	–	
9. Guilt if skipping training	0.46**	0.15**	0.35**	0.28**	0.29**	0.31**	0.26**	0.24**	–
10. Calorie counting during training	0.38**	0.06	0.22**	0.23**	0.32**	0.16**	0.08	0.09*	0.25**

*p < 0.05; **p < 0.01

Hierarchical multiple regression analysis was performed to investigate the effect of (1) gender and age, (2) the number of occasions and the time spent with training, (3) the obsessive aspect of doing sports, (4) health anxiety, and (5) the motivational factors on orthorexia nervosa (see Table 3). Preliminary analyses reported above showed no multicollinearity among the variables. Each model had a significantly better predictive effect than the previous one. The final model included theoretically relevant variables from which the number of training occasions, the time spent with training and the weight control motivation did not predict ON, while the other variables were all statistically significant predictors with the strongest predictors being the social desirability motivation, guilt over skipping training, and health anxiety. Overall, the predictors explained 46% of the variance of orthorexia nervosa.

**Table 3:** Hierarchical regression model of orthorexia nervosa

	*B*	SE	*β*
Step 1 (adjusted *R*^2^ = 0.068)			
Gender	− 0.22	0.05	− 0.18**
Age	0.01	0.00	0.20**
Step 2 (adjusted *R*^2^ = 0.095)			
Gender	− 0.23	0.05	− 0.19**
Age	0.01	0.00	0.20**
Weekly occasions of training	− 0.08	0.03	− 0.13*
Hours spent with training	− 0.02	0.02	0.05
Step 3 (adjusted *R*^2^ = 0.319)			
Gender	− 0.18	0.04	− 0.15**
Age	0.01	0.00	0.12**
Weekly occasions of training	0.04	0.03	0.06
Hours spent with training	− 0.01	0.02	− 0.02
Guilt if skipping training	0.20	0.02	0.36**
Calorie counting during training	0.14	0.02	0.27**
Step 4 (adjusted *R*^2^ = 0.371)			
Gender	− 0.17	0.04	− 0.14**
Age	0.00	0.02	0.09**
Weekly occasions of training	0.08	0.03	0.12**
Hours spent with training	− 0.02	0.02	− 0.04
Guilt if skipping training	0.18	0.02	0.32**
Calorie counting during training	0.13	0.02	0.26**
Health anxiety	0.35	0.05	0.24**
Step 5 (adjusted *R*^2^ = 0.463)			
Gender	− 0.12	0.04	− 0.10**
Age	0.00	0.00	0.09**
Weekly occasions of training	0.04	0.03	0.06
Hours spent with training	− 0.02	0.02	− 0.05
Guilt if skipping training.	0.12	0.02	0.22**
Calorie counting during training	0.09	0.02	0.18**
Health anxiety	0.30	0.05	0.20**
Mood modification	0.08	0.02	0.12**
Weight control	0.05	0.02	0.07
Social desirability	0.14	0.02	0.23**
Healthy lifestyle	0.10	0.03	0.11**

*B* unstandardized regression coefficient, *SE* standard error of B, *β* standardized regression coefficient, *R*^*2*^ proportion of explained variance

*p < 0.05; **p < 0.01

## Discussion

The present study investigated the motivational background of ON with paying particular attention to the role of physical activity and with taking into consideration the motivational features of classical eating disorders. By combining these distinct areas of research, the present study offers a new insight into ON and contributes to the growing area of research on eating disorders.

Increased physical activity seems to play an important role in ON. The results of the present study—i.e., high ON tendency is associated with the number of occasions and the time spent with training—were in accordance with earlier studies showing that people with ON tend to exercise more frequently than the average population [6, 11, 12]. However, when taking into account other variables in the subsequent analyses, the predictive effect of the number of training occasions and the time spent with training disappeared, and the “obsessive” features of sport activities—as the guilt over skipping training and counting calories during training—emerged as strong predictors. Similar tendencies have been found in the restrictive subtype of anorexia nervosa [9]. According to these results, people with high ON tendency might not enjoy the “pleasure side” of physical activity; rather, they might perceive sport activity as a tool for regulating their “needs”, and similar obsessive tendency can appear towards sport as it has been seen in the case of eating. It should be noted that people with ON can be characterized with perfectionist attitude [30]; and this increased perfectionism may explain some features of the abovementioned characteristics of physical exercise as well.

The overlap between ON and obsessive–compulsive disorder (OCD) is widely documented: individuals with ON can be characterized with recurring, intrusive thoughts about healthy eating, as well as with increased concern about infections, contaminations and impurity [31]. Moreover, ON symptom severity is related to OCD symptom severity [32], with potentially similar mechanisms manifesting such as rigidity, a strong need for control, or perfectionism [33]. According to our results, the obsessive mechanisms in ON might extend not only to “healthy” eating, but also to sport activities. It is possible that people with high ON tendency are trying to balance their weak internal control and self-directness skills [23] through external behavioral mechanisms in the form of physical activity.

A detailed analysis of the social desirability motivational factor reveals that people with a higher level of ON tend to reach other people’s respect through healthy eating, and they believe that if they eat ‘pure’, they will receive more positive feedback from the environment. The need for social reinforcement can be explained by earlier results suggesting that openness, awareness and internalization of the Western cultural values about physical fitness and slimness increases the vulnerability to ON [11]. In general, people with ON experience greater intrapersonal distress, have difficulties in social functioning [3] and can be characterized with high social anxiety [11]. It is possible that they attempt to solve their social difficulties and try to become a member of a new community through their eating habits. As reported by Dunn and Bratman [3], individuals with ON attribute a magical role to their diet, which might help them in gaining control over their life and in achieving more success which—in the light of our results—can refer to the social aspect of their lives.

Worrying about health can be adaptive, because it motivates the individual to monitor physical symptoms and promotes self-care and self-awareness behaviors [34]. However, intense fear and anxiety can lead to misinterpretation of normal body signs, which can result in presumption of serious, life-threatening illnesses [35]. To date, no study investigated the role of health anxiety in ON, although previous research showed that people with ON can be featured with high trait anxiety [31] and hypochondrial symptoms [32]. The results of the present study suggest that health anxiety is a strong predictor of ON. Behind the motivation of “pure”, unilateral and high-quality nutrition, people with ON might develop compulsive eating behavior to protect their health, which may include increased monitoring of internal bodily signs. In this regard, ON can be described as a coping mechanism against health anxiety. In many cases, ON symptoms occur after a diagnosis of a disease (e.g., asthma, IBS, allergies), in which the patients have to follow certain rules to eliminate potential irritable components from their diet [36]. Nevertheless, these changes in nutrition can lead to fixations with special diet followers being more likely to be affected by ON [37]. Consequently, the choice of food is not only determined by quality aspects, but also by preventing a given disease.

Based on the healthy eating motivational questionnaire, mood modification was a predictor of ON as well, which suggests that individuals with high ON tendency try to improve/regulate their negative affective states with the help of healthy eating and exercising, which is in line with previous findings [3, 21, 24]. More specifically, individuals with anorexia nervosa and bulimia try to reduce their guilty feelings through dietary restrictions [19]. They have difficulties with emotional regulation [24] and, similar in ON, patients tend to solve intrapersonal and social problems with the help of healthy eating [3].

In sum, we could identify diverse motivational base behind ON, in which the individual overrates and emphasizes different aspects of healthy lifestyle. People with ON attribute a special role to their lifestyle from which they expect far-reaching positive changes in almost every area of their life. The intense focus on health can be defined as a coping style through which the person attempts to reduce anxiety and solve difficulties by an obsessive attitude towards health. It would be beneficial to further investigate whether any examined factors in the present study (obsessive–compulsive features of sport, health anxiety, social desirability) could be added as new topics/items to the existing measuring instruments of ON. According to the reviewed studies [3, 5, 19] and our results, reconstructing and revising the available ON questionnaires are worth considering.

Finally, some limitations of our study need to be mentioned. The results of the present study were based on online data gathering which questions the representative nature of the sample. Although participants were represented in a broad range of age and educational level, the highest proportion of participants were young and highly qualified women. Future studies should focus on the recruitment of more diverse samples to strengthen the generalizability of the findings. The participation in the research was voluntary, thus the increased interest towards the topic of the examination might bias the results. Presumably, women are paying more attention to healthy lifestyle, because self-care and care for others are part of the traditional female role. The correlational design of the present study does not allow causal inferences. A viable next step in ON research could be the use of experience sampling method which could provide important information about the daily fluctuation of ON and the related variables. It would be beneficial to collect information about the type of the exercise as well, because some sport activities (as weight category sports, aesthetic sports) can mean a higher vulnerability to ON. The subject of the present study was the tendency to ON, not orthorexia nervosa as a diagnosis, because ON is not part of the psychiatric nosology system. Therefore, the results cannot be fully transported into ON since no ON diagnosed patients are available yet.

## Conclusion

To summarize, the current study suggests that there is a diverse motivational background behind the tendency of ON. Increased physical activity played a special role, but according to our results individuals with ON highlight the obsessive features (guilt over skipping training, counting calories) of sport activities instead of enjoying the “pleasure side” of it. Moreover, people with ON believe that they can earn others’ respect, improve/regulate their negative affective states, and decrease health anxiety through healthy eating. Therefore, in this case, individuals with ON may expect far-reaching positive outcomes in almost every aspects of their life from healthy eating and sport activities.

## Electronic supplementary material

Below is the link to the electronic supplementary material.

Supplementary material 1 (DOCX 33 KB)
