# Supplementary material for: How the obsession to eat healthy food meets with the willingness to do sports: the motivational background of orthorexia nervosa
Source: Eat Weight Disord. 2019 Feb 6;24(3):465–72. doi: 10.1007/s40519-019-00642-7 (PMC6531400; doi:10.1007/s40519-019-00642-7)
Supplement: Supplementary file 1 — Supplementary material 1 (DOCX 33 KB) [file 40519_2019_642_MOESM1_ESM.docx]

***Online Supplements for:***

**How the obsession to healthy food meets with the willingness to do sports: The motivational aspects of Orthorexia Nervosa**

These online supplements are to be posted on the journal website and hot-linked to the manuscript. If the journal does not offer this possibility, these materials can alternatively be posted on one of our personal websites (we will adjust the in-text reference upon acceptance).

We would also be happy to have some of these materials brought back into the main manuscript, or included as published appendices if you deem it useful. We developed these materials to provide additional information and to keep the main manuscript from becoming needlessly long.

**Appendix 1: Construction of the Motivation for Healthy Behaviors in Orthorexia Nervosa Questionnaire**

In order to assess participants’ underlying motivational background for healthy behaviors, 20 items were generated on the basis of previous case studies and research on orthorexia (Bratman, 1997; Brytek-Matera, 2012; Dunn & Bratman, 2016; Larsen, 2013; Mathieu, 2005; Varga et al., 2014) and other eating disorders (Arcelus, Mitchell, Wales, & Nielsen, 2011; Jánosi & Túry, 2014; Kaye, Frank, & McConaha, 1999; Lavender et al., 2015). Based on their content, these items can be grouped to a total of six factors: mood modification (3 items; Dunn & Bratman, 2016; Larsen, 2013; Lavender et al., 2015), compensation (2 items; Jánosi & Túry, 2014; Kaye et al., 1999), the irrealistic way of thinking about healthy lifestyle (4 items; Bratman, 1997; Mathieu, 2005), healthy lifestyle for weight control (4 items; Arcelus et al., 2011; Brytek-Matera, 2012; Kaye, 2008), social desirability (4 items; Dunn & Bratman, 2016; Varga et al., 2014), and sport (3 items; Herranz Valera et al., 2014; Segura-García et al., 2012; Stochel et al., 2013; Varga et al., 2014).

As the motivational questionnaire was constructed for the purpose of the present study, in the first step of the structural investigation, we performed exploratory factor analysis (EFA) on the 20 items to identify the underlying motivational factors. The sample was used as in the main study. Given that the scales had few response options, the weighted least squares mean-adjusted and variance-adjusted estimator was selected for model estimation, which was found to be more suitable to the ordered-categorical nature of Likert scales with ﬁve or less answer categories relative to maximum-likelihood-based estimation methods (Finney & DiStefano, 2006; Rhemtulla, Brosseau-Liard, & Savalei, 2012; Sass, Schmitt, & Marsh, 2014). In addition, the oblique Geomin rotation was selected as the factors were hypothesized to be interrelated at least moderately. To find the best fitting solution, one to eight-factor models were extracted. After establishing the final solution, confirmatory factor analysis (CFA) was performed on the same sample to verify the structure of the final measurement model. While estimating the same model with EFA and CFA is generally not advised, there are some conditions where it might be acceptable, particularly when one wishes to better understand the data generating process and the factor structure of the construct at hand (Schmitt, Sass, Chappelle, & Thompson, 2018). All factor analyses (EFA and CFA) were conducted in Mplus 8.1 (Muthén & Muthén, 1998-2017).

In evaluating and comparing the alternative models, typical goodness-of-ﬁt indices were examined with their respective thresholds (Hu & Bentler, 1999; Marsh et al., 2005; Marsh, Hau, & Wen, 2004): the comparative ﬁt index (CFI; ≥ .95 for good, ≥ .90 for acceptable), the Tucker–Lewis index (TLI; ≥ .95 for good, ≥ .90 for acceptable), the root mean square error of approximation (RMSEA; ≤ .06 for good, ≤ .08 for acceptable) with its 90% conﬁdence interval. For model selection in EFA, fit indices were taken into account. A model was deemed acceptable when all three fit indices reached at least the criteria for acceptable fit.

A total of eight solutions were tested, ranging from one-factor to eight-factors. Fit indices associated with these models are reported in Table S1. The results indicate that model fit gradually improved with the inclusion of additional factors. These results suggest the one- and two-factor solutions do not fit the data well. While the three-factor model appears to be acceptable, its TLI value is marginally acceptable, and its RMSEA value is less than satisfactory. On the other hand, the four-factor solution reached acceptability on all three indices (CFI > .95, TLI > .90, RMSEA < .08). Eigenvalues also supported the extraction of a four-factor solution. However, we did not wish to select a model solely on the basis of fit indices; thus, we examined the parameter estimates (i.e., factor loadings) associated with the four-factor model as well as the adjacent three- and five-factor solutions as well (see Table S2). Although the three-factor solution had relatively well-defined factors, the addition of a fourth factor resulted in a theoretically meaningful factor. However, the inclusion of a fifth factor resulted in the arbitrary division of one factor into two smaller ones. Moreover, this additional factor was not well-defined by its factor loadings (i.e., all loadings lower than .412), arguing against the need to incorporate a fifth factor. As we aimed to construct a short and reliable scale, we retained two items per factor with the highest factor loadings. Auxiliary confirmatory factor analysis (CFA) was also performed which supported the adequacy of the model as apparent by the satisfactory level of fit indices (CFI = .992, TLI = .984, RMSEA = .060 [90%CI .042-.078]). All four factors were well-defined by their items (λ = .69 to .92, M = .85) and the correlations between the factors were moderate (r = .28 to .59, M = .40). Finally, the model-based omega composite reliability indices (McDonald, 1970) also showed adequate levels of reliability (ω = .738 to .907, M = .840).

The first factor was labeled *mood modification* and referred to experiences when individuals monitor their health because they are in a negative affective state. The second factor was labeled *weight control* and referred to being healthy to achieve the desired weight. The third factor was labeled *social desirability* and refers to being healthy to gain positive feedback and appreciation from the social environment. Finally, the fourth factor was labeled *healthy lifestyle* and encompassed the beliefs that being healthy has positive consequences on one’s life.

**Table S1**

*Fit indices related to measurement models based on Exploratory Factor Analysis*

| Number of factors | χ^2^ | df | CFI | TLI | RMSEA | 90% CI |
| --- | --- | --- | --- | --- | --- | --- |
| 1 | 2971.485* | 209 | .784 | .761 | .134 | .129-.138 |
| 2 | 1989.984* | 188 | .859 | .827 | .114 | .109-.118 |
| 3 | 1087.915* | 168 | .928 | .901 | .086 | .081-.091 |
| **4** | **771.658*** | **149** | **.951** | **.925** | **.075** | **.070-.080** |
| 5 | 549.463* | 131 | .967 | .942 | .066 | .060-.071 |
| 6 | 327.694* | 114 | .983 | .966 | .050 | .044-.057 |
| 7 | 241.177* | 98 | .989 | .974 | .044 | .037-.052 |
| 8 | 152.232* | 83 | .995 | .985 | .034 | .025-.042 |

*Note.* χ^2^: Chi-square test; df: degrees of freedom; CFI: comparative ﬁt index; TLI: Tucker-Lewis index; RMSEA: root mean square error of approximation; 90% CI: 90 % confidence interval associated with RMSEA; Bolded letters indicate the final model.; **p* < .01.

**Table S2**

*Standardized factor loadings for the three-factor, the four-factor, and the five-factor EFA models*

|  | Three-factor model | | | Four-factor model | | | | Five-factor model | | | | |
| --- | --- | --- | --- | --- | --- | --- | --- | --- | --- | --- | --- | --- |
|  | 1 | 2 | 3 | 1 | 2 | 3 | 4 | 1 | 2 | 3 | 4 | 5 |
| ITEM 1 | .486* | -.042 | .295* | .452* | -.029 | .285* | .115 | .509* | .040 | -.029 | .147* | .136* |
| ITEM 2 | .880* | .016 | -.163* | **.849*** | .068 | -.080 | -.053 | .791* | .412* | .103* | -.043 | -.081 |
| ITEM 3 | .816* | -.109* | .000 | **.792*** | -.061 | -.040 | .084* | .742* | .400* | -.033 | .011 | .034 |
| ITEM 4 | .462* | .223* | .297* | .424* | .232* | .290* | .125* | .539* | -.128 | .191* | -.026 | .260* |
| ITEM 5 | .444* | -.003 | .475* | .418* | .016 | .273* | .319* | .495* | -.019 | -.017 | .075 | .381* |
| ITEM 6 | .506* | .012 | .286* | .464* | -.006 | .519* | -.082 | .665* | -.313* | -.046 | .070 | .033 |
| ITEM 7 | .347* | .189* | .089 | .300* | .174* | .407* | -.209* | .437* | -.260* | .160* | .059 | -.086 |
| ITEM 8 | .026 | .257* | .487* | -.049 | .248* | **.593*** | .046 | .067 | -.315* | .236* | .445* | .084 |
| ITEM 9 | .015 | .004 | .640* | -.072 | -.035 | **.728*** | .116 | -.010 | -.213 | -.001 | .827* | -.022 |
| ITEM 10 | .083 | .007 | .721* | .023 | -.004 | .541* | .362* | .058 | -.010 | .024 | .589* | .303* |
| ITEM 11 | .256* | .508* | .199* | .235* | .512* | .128* | .154* | .285* | -.037 | .471* | -.013 | .278* |
| ITEM 12 | .156 | .748* | -.032 | .139* | .750* | -.025 | .039 | .102* | .157* | .710* | .104 | .111* |
| ITEM 13 | .059 | .910* | -.128* | .034 | **.894*** | .013 | -.089* | -.041 | .198* | .895* | .259* | -.057* |
| ITEM 14 | .064 | .775* | .020 | .030 | **.760*** | .151* | -.052 | .036 | .016 | .722* | .213* | .036 |
| ITEM 15 | -.123 | .417* | .389* | -.111* | .422* | -.004 | .430* | -.094 | -.080 | .353* | -.010 | .506* |
| ITEM 16 | -.128 | .688* | .265* | -.128* | .683* | .012 | .303* | -.114* | -.094 | .621* | .005 | .417* |
| ITEM 17 | .045 | .363* | .261* | .047 | .368* | .022 | .284* | .035 | .060 | .337* | .089 | .313* |
| ITEM 18 | .098 | .611* | .195* | .100* | .624* | -.050 | .284* | .106* | .030 | .572* | -.076 | .396* |
| ITEM 19 | -.049 | .000 | .948* | .004 | .030 | .057 | **.927*** | .019 | .013 | -.049 | .083 | .931* |
| ITEM 20 | -.020 | .157* | .765* | .041 | .198* | .008 | **.749*** | .059 | -.002 | .117* | -.004 | .795* |
| ITEM 21 | .030 | -.597* | .243* | .042 | -.576* | -.011 | .241* | -.015 | .214* | -.540* | .160 | .090 |
| ITEM 22 | .016 | -.436* | .538* | .032 | -.414* | .048 | .521* | -.019 | .195* | -.399* | .208* | .405* |

*Note.* Grayscale items are adequate indicators of their factors as evidenced by the factor loadings being higher than .500 (as suggested by Morin, Myers, & Lee, 2018).; Bolded items were selected as final ones in the four-factor model.; **p* < .05.

**Hungarian and English versions of the Motivation for Healthy Behaviors in Orthorexia Nervosa Questionnaire**

|  | **Hungarian version** | **English version** |
| --- | --- | --- |
| Title | Motiváció az Egészséges Viselkedésre Orthorexia Nervosában Kérdőív | Motivation for Healthy Behaviors in Orthorexia Nervosa Questionnaire |
| Instructions | Kérjük, válaszoljon a következő állításokra aszerint, hogy mennyire jellemző Önre! Nincsenek jó vagy rossz válaszok. Kérjük, minden kérdésre őszintén válaszoljon! | Indicate how much the following statements characterize you! There are no right or wrong answers. Reply as honestly as possible. |
| Rating scale | 1 – teljes mértékben igaz  2 – többnyire igaz  3 – többnyire nem igaz  4 – egyáltalán nem igaz | 1 – completely true  2 – true  3 – not true  4 – not true at all |
| Item 1 (mood modification) | Ha rossz hangulatban vagyok, jobban odafigyelek az egészséges étkezésre. | I monitor more closely to eat healthily when I’m in a bad mood. |
| Item 2 (weight control) | Egészséges ételek fogyasztásával elérhetem a kívánt testsúlyt. | I can reach the ideal body weight by consuming healthy food. |
| Item 3 (social desirability) | Egészséges étkezéssel kiválthatom mások megbecsülését. | I can evoke others’ appreciation by eating healthy. |
| Item 4 (healthy lifestyle) | Úgy gondolom, hogy az egészséges táplálkozás és testedzés javíthat a mindennapi közérzetemen. | I think that healthy eating and exercising can improve the way I feel daily. |
| Item 5 (mood modification) | Ha rossz hangulatban vagyok, jobban odafigyelek a rendszeres testmozgásra. | When I’m feeling down, I pay more attention to exercising regularly. |
| Item 6 (weight control) | Rendszeres testmozgással elérhetem a kívánt testsúlyt. | Through regular exercising I can achieve the desired weight. |
| Item 7 (social desirability) | Ha egészségesen táplálkozom, több pozitív visszajelzést kapok a környezetemtől. | If I consume healthy food, I receive more positive feedback from my social environment. |
| Item 8 (healthy lifestyle) | Úgy érzem, hogy az egészséges táplálkozás és testedzés testi és lelki szinten is egészséget eredményez. | I feel that healthy eating and exercising result in mental and physical health. |
